# Metabolic Shifts Precede Cognitive Decline in the Male hAß‐KI Alzheimer's Mouse Model

**DOI:** 10.1096/fj.202602435RR

**Published:** 2026-08-28

**Authors:** Evelin Melekh, Emma L. Cook, Julia G. Stante, Amélie A. T. Marais, Ahmad Mohammad, Shawn M. Beaudette, Wendy E. Ward, Val A. Fajardo, Rebecca E. K. MacPherson

**Affiliations:** ^1^ Department of Health Sciences Brock University St. Catharines Ontario Canada; ^2^ Department of Kinesiology Brock University St. Catharines Ontario Canada; ^3^ Centre for Bone and Muscle Health Brock University St. Catharines Ontario Canada; ^4^ Centre for Neurosciences Brock University St. Catharines Ontario Canada

**Keywords:** Alzheimer's disease, cognitive decline, hAß‐KI, metabolic dysfunction, sex differences

## Abstract

A major barrier in AD research is the lack of animals that recapitulate sporadic AD, which accounts for most human cases. Recently, a novel mouse model (hAβ‐KI) was developed by introducing a three‐point mutation that humanizes the beta‐amyloid peptide. These mice exhibit age‐dependent cognitive decline and beta‐amyloid accumulation. However, limited research has been conducted related to AD risk factors and sex‐specific responses in this model. Male and female control and hAß‐KI mice were assessed at ages 4, 8, 10, 12 and 15 months. Body composition (bone mineral density, lean and fat mass) and metabolic function (respiratory exchange rate, energy expenditure, and activity levels) were evaluated, in addition to insulin tolerance testing (ITT). An open field test (OFT) was performed to evaluate anxiety‐like behaviors, and novel object location (NOLT) and recognition tests (NORT) were conducted to measure cognitive impairment at endpoint. With aging, the male hAß‐KI mice exhibited increased body and fat mass %, decreased lean mass %, decreased activity, and impaired glucose handling prior to confirmed cognitive impairment by NORT and NOLT. In contrast, the female hAß‐KI did not display similar differences compared to controls. These findings support the utility of this model as a valuable tool for investigating systemic risk factors related to sporadic AD, and the absence of similar differences in the females highlights the importance of considering sex as a variable in AD pathology.

## Graphical abstract

A major barrier in Alzheimer's disease (AD) research is the lack of animals that recapitulate sporadic AD. The humanized amyloid‐beta knock‐in mice (hAß‐KI) exhibit age‐dependent cognitive decline and beta‐amyloid accumulation. However, limited research has been conducted related to AD risk factors and sex‐specific responses. This study demonstrates that male hAß‐KI mice developed early, metabolic and body composition changes that preceded cognitive decline, supporting this model as a valuable tool for studying systemic risk factors related to sporadic AD. The absence of similar differences in the females highlights the importance of considering sex as a variable in AD pathology.

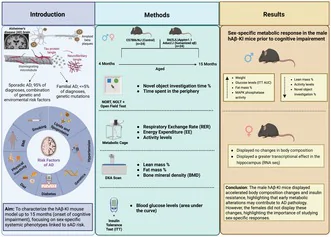

## 1 Introduction

Alzheimer's disease (AD) is a progressive neurodegenerative disease defined primarily by deteriorating memory and cognitive decline. AD is the most common type of dementia, accounting for approximately 60% of all dementia cases and is the most prevalent form of dementia in individuals aged 65 years and older [1, 2, 3]. The incidence for this disease continues to increase worldwide; in 2011, the estimated prevalence for AD was 24 million, and projections suggest that this number could reach 48 million by 2030 [1]. Given this information, understanding the factors that contribute to AD progression is crucial for guiding and developing strategies to reduce AD risk throughout aging.

Familial AD (fAD), also known as early onset AD, typically occurs prior to 60 years of age with symptoms often appearing at approximately 40–50 years old [1, 2, 3]. This form of AD represents ~5% of all AD cases and is caused by genetic mutations in predominantly three genes: amyloid precursor proteins (APP), presenilin 1 (PSEN1), and presenilin 2 (PSEN2) [1, 2, 3]. In contrast, sporadic AD (sAD), also known as late‐onset AD, typically occurs after the age of 65 and represents ~95% of diagnosed AD cases. sAD is a multifactorial disease, involving a combination of non‐modifiable and modifiable risk factors. Non‐modifiable risk factors for sAD include age, Apolipoprotein E4 (APOE4) carrier status, and biological sex, with females showing a higher lifetime risk of developing AD compared to males [1]. Modifiable risk factors associated with AD include poor diet, physical inactivity, midlife obesity, hypertension, hearing loss, and smoking [4]. Furthermore, conditions such as hypercholesterolemia, hyperinsulinemia, increased serum proinflammatory cytokines, and altered adipokines also increase the risk of developing sAD [1, 4, 5].

Preclinical research is essential to understanding the mechanistic disease processes involved in AD and for testing potential therapeutic strategies in controlled experimental models before the translation to human studies. The landscape of preclinical AD research is undergoing a critical shift; for decades, the field has predominantly relied on transgenic overexpression models on conventional inbred mouse backgrounds, particularly C57BL/6J, to investigate AD pathogenesis and evaluate candidate therapeutics. While these models have yielded invaluable mechanistic insights, their limited genetic diversity, supraphysiological expression of familial AD genes (e.g., APP, PSEN1), and poor translation to human clinical outcomes have exposed fundamental limitations [6]. There is an urgent need for more representative and scalable models that better capture the complex, polygenic, and late‐onset nature of sAD as it occurs in humans.

Recently, Baglietto et al. developed a humanized mouse model of AD (referred to as hAß‐KI in this manuscript) in which a 3‐point mutation was introduced to knock‐in the human wildtype Aß peptide, thereby avoiding the introduction of fAD mutations and making this model potentially relevant for sAD [7]. These hAß‐KI mice demonstrate aging‐associated cognitive decline, with novel object recognition test (NORT) deficits emerging at 14 months and persisting to 22 months (the age at which the study was completed) [7]. In the hippocampus, these mice demonstrated a shift from soluble levels of Aβ to insoluble Aβ levels with aging that peaked at 18–22 months [7]. This was consistent with a progressive, slowly developing amyloidopathy. In vitro seeding assays further demonstrated that hAß‐KI brains displayed accelerated Aβ aggregation similar to the positive control of the fAD mouse model sample, despite the absence of extracellular plaque deposits in vivo [7]. These findings suggest that this model recapitulates early pre‐plaque stages of human‐like Aß dysregulation, and that additional environmental or metabolic challenges, such as a western diet, may be required to trigger plaque formation [7]. Since the development of this model, other researchers have characterized spatial memory deficits using the Barnes maze [8], mitochondrial function [9], synaptic alterations [8], neuroinflammation, and introduced interventions with a focus on altering markers related to mitochondrial health and histone acetylation [8, 10]. These studies reinforce the utility of this mouse model for studying cellular vulnerability in a sporadic‐like context; however, to date no studies have characterized systemic phenotypes in these mice, such as changes in metabolic factors prior to the onset of memory deficits, and limited research on sex specific responses has been explored. Given the strong epidemiological association between metabolic dysfunction and sAD, we hypothesize that metabolic dysfunction emerges prior to cognitive impairment.

The objective of this study was to extend the characterization of the hAß‐KI mouse model up to the onset of cognitive impairment (15 months), with a specific focus on systemic phenotypes linked to sAD risk. We therefore evaluated established AD‐related risk factors, including body composition (lean and fat mass, bone mineral density) and metabolic outcomes (insulin resistance, energy expenditure (EE), activity levels), and examined how these variables change with age. Given the well‐established influence of biological sex on AD risk and progression, sex‐specific responses were also analyzed. A detailed characterization of these peripheral and metabolic features in hAß‐KI mice will further establish the utility of this model as a preclinical tool that mirrors age‐related metabolic changes that precede sAD. These results may reveal windows of opportunity for interventions aimed at preventing or slowing the progression of sAD.

## 2 Methods

### 2.1 Animals and Study Design

Animal breeding and experimental protocols for the hAß‐KI colony were approved by the Brock University Animal Care Committee (#23‐08‐02) and followed the guidelines of the Canadian Council of Animal Care. Male and female B6(SJL)‐*App* ^*tm1.1Aduci*^/J (hAß‐KI) and C57BL6/NJ (control) mice were purchased from the Jackson Laboratories to establish an in**‐**house animal colony. Heterozygous hAß‐KI mice were bred to produce wild‐type (APP^−/−^; 25% of all offspring), APP heterozygous mutant (APP^−/+^; 50% of all offspring), and APP homozygous mutant (APP^+/+^; 25% of all offspring). Mice were screened for the desired genotype using protocols outlined by Jackson Laboratories (stock number: 030898), and the primers used are listed in the supplemental section (Table S1A).

For the duration of the whole study, the mice were single‐housed to avoid fighting‐related injuries that could occur due to the prolonged duration of the study, kept on a 12/12‐h light/dark cycle (lights on at 8 am), and had ad‐libitum access to food and water (standard mouse chow diet, 2014 Tekland global, 14% protein rodent maintenance diet, Harlan Tekland, Mississauga). Body mass was measured weekly, and monthly averages were calculated from four consecutive weekly measurements per mouse to assess changes between the control and hAß‐KI mice.

A total of 48 mice (*n* = 24, male and female, *n* = 12/sex/genotype) B6(SJL)‐*App* ^*tm1.1Aduci*^/J and C57BL6/NJ control littermate mice were utilized. In vivo repeated measurement testing at 4, 8, 10, 12, and 15 months of age was performed until reported cognitive impairment based on the Baglietto et al. article [7]. At each of these timepoints, body composition (fat mass, lean mass, BMD) was measured via iNSiGHT Small Animal DXA Scanner, metabolic outcomes (EE, respiratory exchange ratio (RER), activity levels) were measured via Promethion Metabolic Caging System, and insulin tolerance was measured. At endpoint, an open field test (OFT) was performed to evaluate anxiety‐like behaviors, and novel object recognition and novel object location (NOLT) testing were performed to confirm cognitive impairment.

### 2.2 Open Field, Novel Object Recognition and Location Tests (OFT, NORT, NOLT)

For all testing, overhead videos were recorded (30 Hz; Resolution 1920 × 1080 pixels) using a Go Pro Hero 8. The NORT and the NOLTs were performed in three stages: (1) a habituation phase (which acted as the OFT), (2) a familiarization phase, and (3) two testing phases. For all phases, mice were released in the same corner of the arena, and the arenas were sanitized with a peroxigard solution between each phase and mouse. On the day prior to performing the NOLT and NORT, the habituation phase was performed, which consisted of a 10‐min period where mice explored an empty arena (40 × 40 cm) to investigate any anxiety‐like behaviors. The following day, the familiarization phase was performed, which involved mice exploring two identical objects of similar sizes (A1 and A2) for a period of 10‐min. The mice returned to their home cages for an inter‐trial retention delay of 1 h or 4 h. Once the inter‐trial period ended, the mice returned to the arena for the first test phase (NOLT), where one of the objects was moved to a new location (A2), while A1 remained in the same location, and the mice explored for 10 min. Following this, one of the objects (A1) was then replaced with a novel object (NORT), and again the mice explored for 10 min.

Mouse tracking and anatomical key points were extracted using Deeplabcut (DLC v2.3.5). Training data was derived from the manual labelling of 20 distinct frames extracted (k‐means clustering) from 10 input videos with key points located on the ears, base of tail, and the nose of each mouse in the video. Following labeling, a deep neural network was trained (resnet_152) to automatically extract the pose of anatomical key points across all videos. The mean training and testing error of the model was 3.15 and 3.45 pixels, respectively. Time‐varying anatomical key point data were subsequently transferred to MATLAB for further analysis (version 2024b). Object investigation % was derived through a set object radius which was determined based on the largest object used in each trial test. The same object interaction radius was implemented for all objects. All mice needed to be within the target radius and facing the object for an interaction to occur [11, 12, 13]. The object investigation % was calculated as (time spent with novel object) / (sum of total object investigation time) × 100. Results under the threshold of 15% were excluded from the analysis to exclude mice that did not investigate the objects for a substantial amount of time during the testing period.

### 2.3 Metabolic Caging

Mice were single housed in the Promethion high‐definition behavioral phenotyping cages (Sable Systems, North Las Vegas, NV) for 48 h. This system allowed for real‐time behavior analysis of mice in a home‐cage setting and measured EE, RER, and cage activity. The data was recorded for the full 48‐h period; however, only the final 24 h were analyzed to allow for acclimatization to the new environment during the first 24 h.

### 2.4 Dual‐Energy X‐Ray Absorptiometry (DXA) Scanning

A small animal DXA scanner (OsteoSys InSIGHT, Scintica Instrumentation, ON, Canada) was used to non‐invasively measure body composition (lean mass %, fat mass %, BMD). The mice were anesthetized ~3 min for the duration of the scan ~ using vaporized isoflurane (2.5% isoflurane and 250 mL flow) and they were then returned to their home cage to recover. Lean and fat mass (%) were calculated as total lean or fat mass divided by total body mass multiplied by 100 (e.g., 30 g of lean mass in 60 g mouse, 30/60 × 100 = 50%). The change (Δ) in lean and fat mass % were calculated by subtracting the baseline by the endpoint value.

### 2.5 Insulin Tolerance Testing (ITT)

Whole body insulin sensitivity was measured using intraperitoneal insulin tolerance tests (0.75 U/kg of insulin). Blood glucose measures from the snip of the tail were taken at 0, 15, 30, 60, and 120 min after the insulin injection with the use of a hand‐held glucometer (Freestyle Lite, Abbott). Plots of the average changes in glucose over time were made for each group, and the total area under the curve (AUC) was calculated.

### 2.6 Tissue Collection

At 15 months of age, the mice were anesthetized using vaporized isoflurane and euthanized via cervical dislocation following an intracardiac puncture using a 27‐gauge syringe. The blood was placed on ice ~30 min and then centrifuged for 8 min at 8000 ***g*** to collect the serum. The prefrontal cortex (PFC) and hippocampus (HIP) were dissected, snapped‐frozen in liquid nitrogen, and stored in −80°C.

### 2.7 RNA Sequencing

The HIP and PFC (included in supplemental section) from the hAß‐KI mice and control mice (*n* = 3 per group) were homogenized in 1 mL of TRIzol Reagent (15596026, ThermoFisher Scientific, MI, USA) and RNA was extracted using the Qiagen RNeasy Kit (cat: 74104). Total RNA sequencing with purified mRNA was performed by Novogene (Novogene Corporation Inc., Sacramento, CA, USA) using NovaSeqXPlus. Differentially expressed genes (DEGs) between *hA*ß*‐KI Female/Control Female*, and *hAß‐KI Male/Control Male* were identified (log2 fold‐change |log2FC| > 0.32, *p <* 0.05) by Novogene using DESeq2 software, which improves variance estimation, allowing for more robust results even with a small sample size [14]. Statistically significant DEGs were subjected to GO and KEGG pathway enrichment analyses (based on adjusted *p* < 0.05 after Benjamini‐Hochberg (BH) correction). All up and downregulated GO and KEGG pathways were reported.

### 2.8 Serum Analysis

Serum samples were extracted from 4 control wild‐type and 6 hAß‐KI mice at 15 months of age. These samples were sent to Olink as part of their T48 Mouse Pilot Program, which allows for high sensitivity detection of 43 mouse‐derived cytokines, chemokines, and other immune‐related proteins. This assay uses proximity extension assay technology where oligonucleotides were labeled with an antibody probe pair which allowed them to bind to their respective target protein in the sample. Following this, PCR was performed, and the sequences were amplified and detected using real‐time PCR. The results were reported in pg/mL and analyzed using principal component analysis (PCA) to identify patterns of immunological variation between the control and the hAß‐KI mice.

### 2.9 Statistical Analysis

Statistical analyses were performed using GraphPad Prism 10.1.1 (Boston, MA), with standard deviation and the mean reported. All outcomes were analyzed separately by sex to evaluate sex‐specific responses, in accordance with the CIHR institute for Gender and Health Core Competency Module for Sex and Gender in Biomedical Research [15].

For group comparisons, two‐way ANOVA (with genotype and age as factors) or an unpaired *t*‐test analyses were conducted, stratified by sex. Significance was determined at *α* = 0.05.

Linear regression analyses (split by sex) were conducted to evaluate the relationship between the NOLT and NORT cognitive scores and other outcomes such as RER, EE, BMD, change in body, lean, and fat mass. The slope, standard deviation, *R* ^2^, and *p*‐value for deviation of the slope from zero were reported in Table 1. Outcomes with a *p*‐value of 0.051–0.150 were classified as trends and were therefore reported.

For serum cytokine analysis, a PCA was performed, however, due to the small sample size, only three biomarkers (IL6, HGF, IL1a) were selected for analysis. PCA was conducted in SPSS using a correlation matrix and z‐score variables. Prior to analysis, assumptions such as the Kaiser‐Meyer‐Olkin measure, Barlett's test of sphericity and inspection of determinant were evaluated. Components were extracted using the Kaiser criterion (eigenvalues > 1), and variables with correlation coefficient below 0.3 were excluded due to insufficient shared variance.

## 3 Results

### 3.1 Male hAß‐KI Mice Demonstrate Faster Weight Gain, Lean Mass Loss, and Fat Mass

**FIGURE 1:**
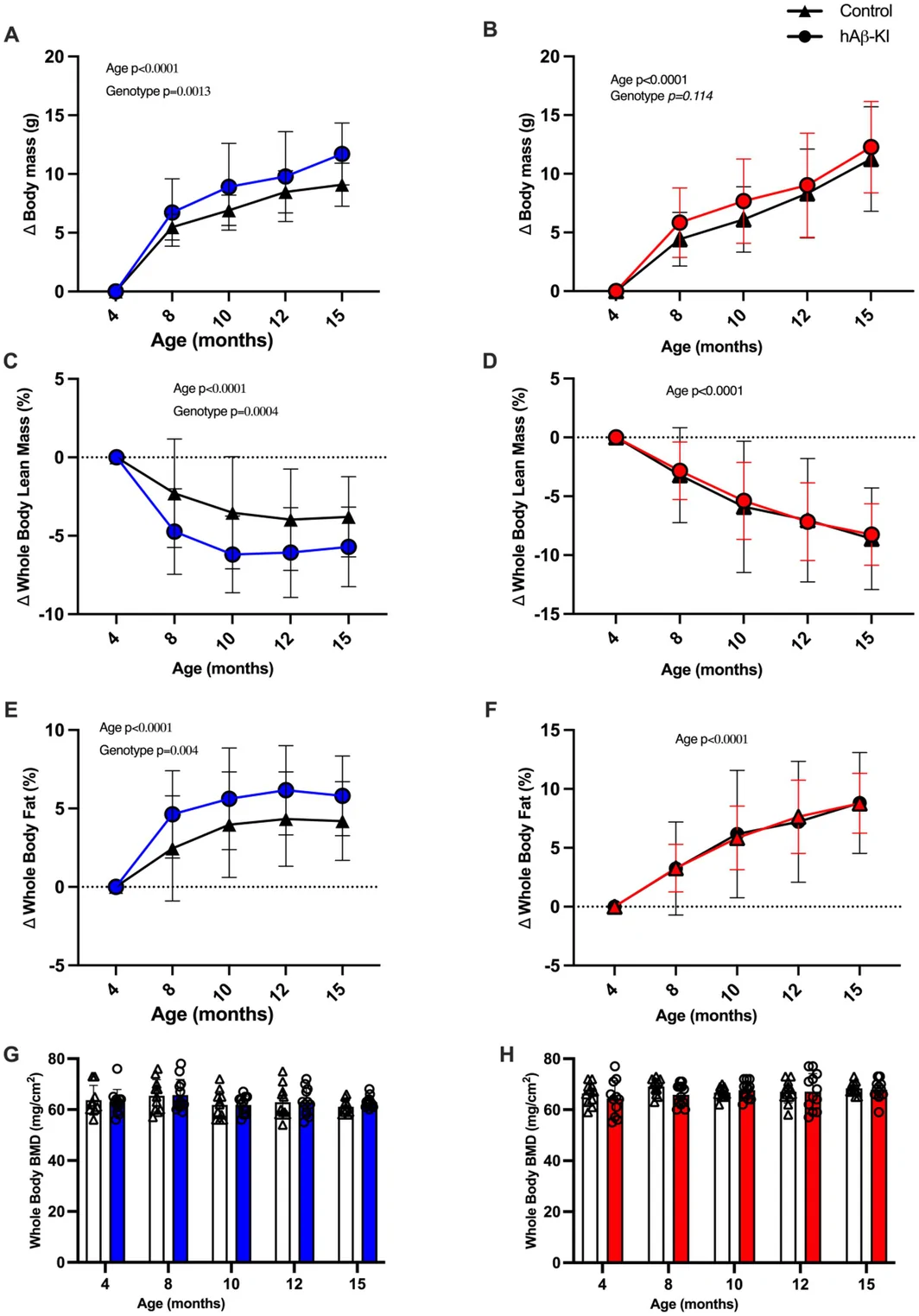
Longitudinal body composition changes in male and female, control and hAß‐KI mice from 4 to 15 months of age. Triangular symbols represent control mice, and circular symbol represent hAß‐KI mice, the blue symbol represent male mice, red symbols represent female mice. (A, B) change in body mass (g) across aging, (C, D) change in lean mass % , (E, F) change in body fat % , (G, H) whole body bone mineral density (mg/cm^2^). *n* = 12/genotype/sex, total (*n* = 48) per timepoint. Mean and +/− SD were reported using a Two‐Way ANOVA. *p* < 0.05 was considered statistically significant.

Both the control and hAß‐KI mice gained body mass with aging (*p* < 0.0001) as expected; however, the male hAß‐KI mice gained more compared to their control counterpart (genotype *p* = 0.0013), while a trend was seen for the main effect of genotype (*p* = 0.114) with the female hAß‐KI mice having a higher gain in body mass compared to their control counterpart (Figure 1A,B). A main effect for genotype (*p* = 0.0004) and age (*p* < 0.0001) were noted in the males for change in lean mass, indicating the hAß‐KI male mice were losing more lean mass % compared to the controls throughout aging, while a main effect of age (*p* < 0.0001) was reported in the females with no genotype differences, indicating that the females were losing lean mass % as they aged (Figure 1C,D). There was a main effect of genotype (*p* = 0.004) and age (*p* < 0.0001) for change in fat mass % in the males, indicating the hAß‐KI male mice gained more fat during aging, while the females had a main effect of age (*p* < 0.0001) with no genotype differences (Figure 1E,F). Lastly, there were no main effects or interactions for genotype or age with whole body BMD for the male and female mice (Figure 1G,H).

### 3.2 Male hAß‐KI Mice Exhibit Lower Activity Levels and Increased Insulin Resistance

**FIGURE 2:**
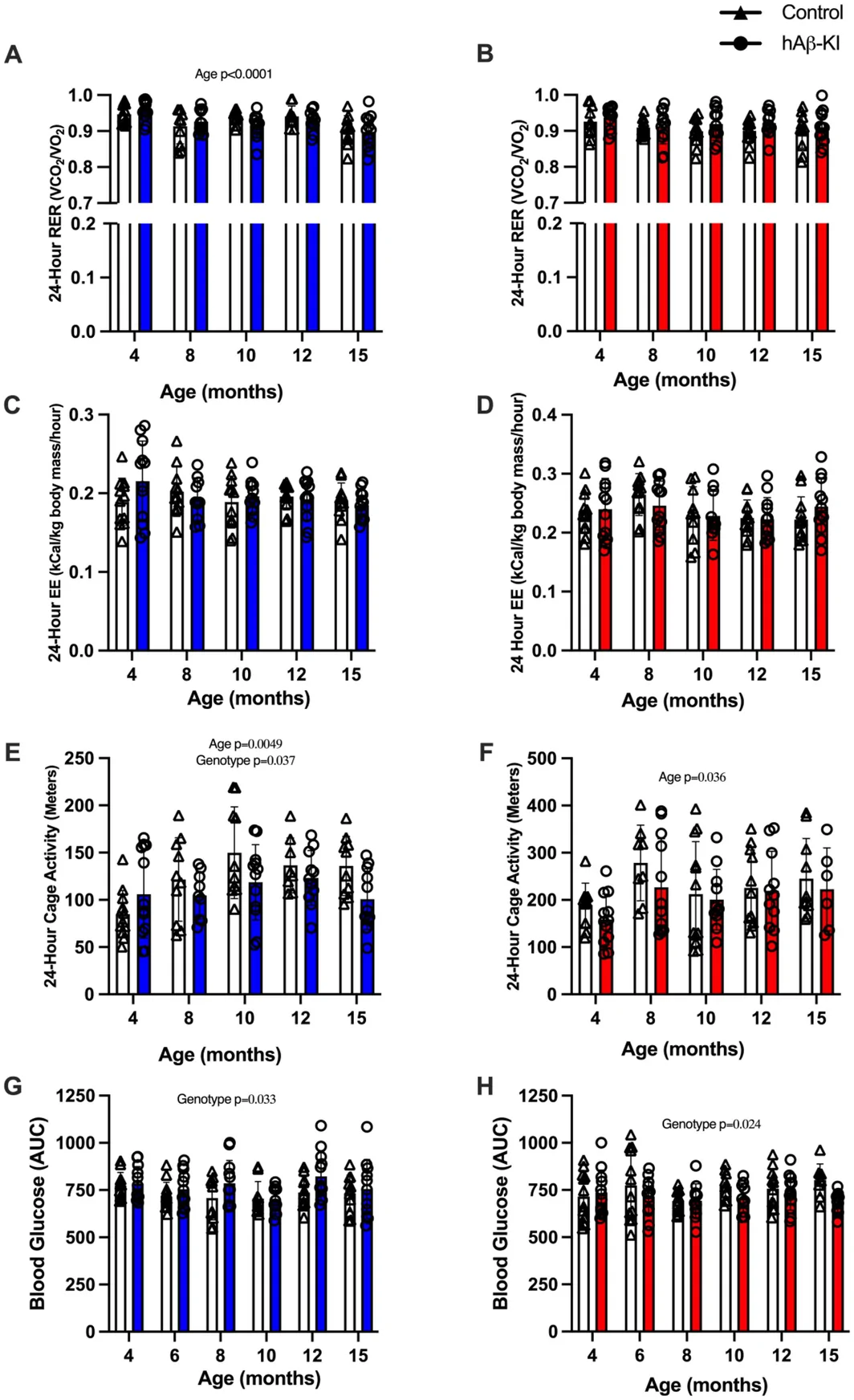
Longitudinal metabolic outcomes in male and female, control and hAß‐KI mice from 4 to 15 months of age. Triangular symbols represent control mice, and circular symbol represent hAß‐KI mice, the blue symbol represent male mice, red symbols represent female mice. (A, B) 24‐h respiratory exchange rate (RER, VCO_2_/VO_2_) across aging, (C, D) 24‐h energy expenditure (EE, kcal/h/kg body mass) across aging, (E, F) 24‐h cage activity (meters) across aging, (G, H) area under the curve (AUC) for blood glucose levels for insulin tolerance test (ITT). *n* = 12/genotype/sex, total (*n* = 48) per timepoint. Mean and ± SD were reported using a Two‐Way ANOVA. *p* < 0.05 was considered statistically significant.

There was a main effect of age for RER in the males, which showed that as the male mice aged their RER decreased, while no main effects were noted in the females (Figure 2A,B). No main effects were noted in the energy expenditure for both sexes (Figure 2C,D). For cage activity, the males displayed a main effect for age (*p* = 0.004) and genotype (*p* = 0.037). The male mice exhibited the most activity at 10 and 12 months of age; however, the male hAß‐KI mice had lower activity levels compared to control counterparts (Figure 2E). The females also had a main effect of age (*p* = 0.036) (Figure 2F), indicating they moved most at 8 months of age; however, there was no main effect of genotype. For the insulin tolerance test, the male hAß‐KI mice had a higher blood glucose area under the curve compared to their control counterparts (*p* = 0.033) (Figure 2G), while the female hAß‐KI mice showed an opposite effect with lower blood glucose area under the curve compared to their control counterparts (Figure 2H).

### 3.3 Spatial and Recognition Memory Is Linked to Metabolic and Body Composition Outcomes

**FIGURE 3:**
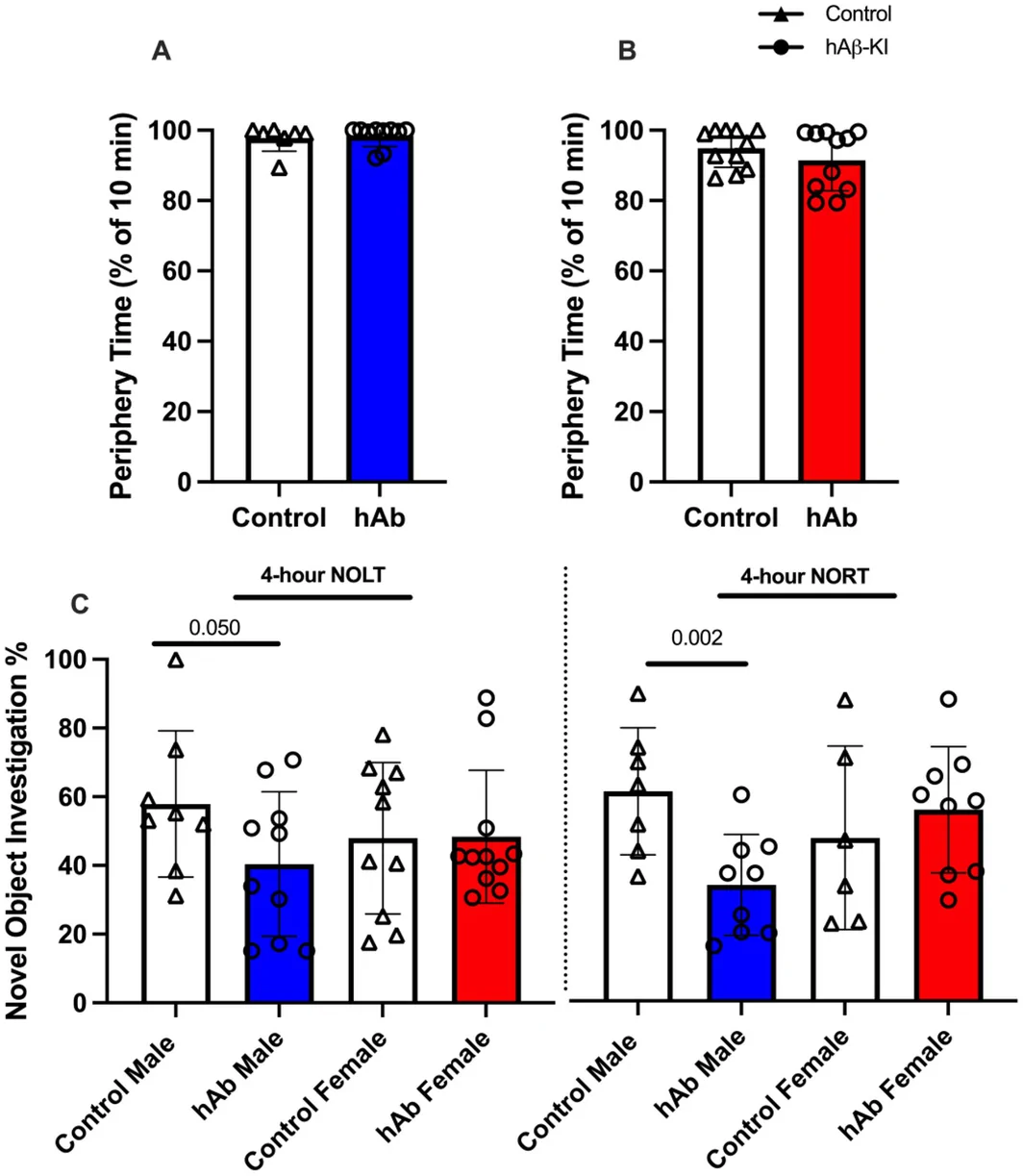
Anxiety‐like behavior and novel object investigation tests in male and female, control and hAß‐KI mice at 15 months of age. Triangular symbols represent control mice, and circular symbol represent hAß‐KI mice, the blue symbol represent male mice, red symbols represent female mice. (A, B) open field test—time spent in the periphery of the arena (% of time out of 10 min), (C) novel object investigation % for 4‐h NOLT and NORT. *n* = 12/genotype/sex, total (*n* = 48) per timepoint. Mean and ± SD were reported using an unpaired t‐test. *p* < 0.05 was considered statistically significant.

Behavioral and memory testing was conducted at 15 months of age. Open field testing found no differences between the groups in the time spent in the periphery of the arena for both male and female mice (Figure 3A,B). Male hAß‐KI mice displayed lower percent investigation time in both the NOLT (*p* = 0.050) and NORT (*p* = 0.002) compared to their control counterparts; however, this was not observed in the female hAß‐KI mice (Figure 3C).

In addition to the cognitive testing, linear regression analysis within the hAß‐KI mice (split by sex) was performed on all outcomes to determine their potential influence on NORT and NOLT scores (Table 1). In the male hAß‐KI mice, body mass was found to have a positive association on NORT, indicating that hAß‐KI mice with higher body mass were found to have higher NORT scores; however, this relationship did not reach statistical significance (*p* = 0.075). RER and NOLT scores in the male hAß‐KI mice were negatively associated, for every unit decrease in RER, there was a concomitant increase in NOLT scores (*p* = 0.023). Activity levels and NOLT scores in the male hAß‐KI mice were positively associated, for every unit increase in ped meters, there was a significant 0.984 unit increase in NOLT scores (*p* = 0.033).

**TABLE 1:** Associations between metabolic measures and NORT and NOLT cognitive tests in the hAß‐KI mice assessed using regression analysis.

Outcomes	NORT		NOLT	
	Male hAß‐KI Slope, SD, *R* ^2^, *p*	Female hAß‐KI Slope, SD, *R* ^2^, *p*	Male hAß‐KI Slope, SD, *R* ^2^, *p*	Female hAß‐KI Slope, SD, *R* ^2^, *p*
Δ Body mass	0.213, ±0.102, 0.382, *p = 0.075*	−0.142, ±0.077, 0.328, *p = 0.106*	0.028, ±0.102, 0.009, *p* = 0.791	0.036, ±0.079, 0.022, *p* = 0.660
Δ Lean mass	−0.080, ±0.063, 0.185, *p* = 0.247	0.074, ±0.055, 0.205, *p* = 0.220	0.056, ±0.045, 0.159, *p* = 0.253	0.032, ±0.045, 0.052, *p* = 0.498
ΔFat mass	0.084, ±0.064, 0.199, *p* = 0.228	−0.097, ±0.048, 0.364, *p = 0.085*	−0.053, ±0.046, 0.143, *p* = 0.280	−0.018, ±0.044, 0.018, *p* = 0.686
BMD	−0.008, ±0.060, 0.002, *p* = 0.896	−0.025, ±0.086, 0.011, *p* = 0.780	0.023, ±0.045, 0.031, *p* = 0.622	0.109, ±0.057, 0.285, *p = **0.090 ***
EE	−0.0001, ±0.0004, 0.013, *p* = 0.764	0.001, ±0.0008, 0.208, *p* = 0.216	0.000006, ±0.0003, 0.00004, *p* = 0.985	−0.0005, ±0.0006, 0.065, *p* = 0.447
RER	−0.001, ±0.001, 0.093, *p* = 0.422	0.0004, ±0.001, 0.0253, *p* = 0.6825	−0.001, ±0.0006, 0.495, ***p* = 0.023**	−0.0002, ±0.0007, 0.014, *p* = 0.720
Ped Meters	−0.264, ±0.692, 0.023, *p* = 0.716	−0.538, ±3.73, 0.004, *p* = 0.891	0.984, ±0.372, 0.499, ***p* = 0.033**	0.813, ±1.488, 0.040, *p* = 0.601

*Note:* Slopes, standard deviation (SD), coefficients of determination (*R* ^2^), and *p*‐values were reported for each sex and cognitive outcome. All significant *p*‐values (< 0.05) were bolded, and trending *p*‐values were italicized.

In the female hAβ‐KI mice, body mass and NORT scores were negatively associated, such as lower body mass corresponded with higher NORT scores, however, this relationship was not statistically significant (*p* = 0.106). Additionally, a negative association with NORT scores and Δ in fat mass, indicated that female mice who gained less fat mass had higher NORT scores, however, this was only trending to significance (*p* = 0.085). Finally, a positive association approaching significance was found in the female hAß‐KI, whereby higher BMD was found in female hAß‐KI mice with higher NOLT scores (*p* = 0.090).

### 3.4 Hippocampus RNA Seq Analysis

**FIGURE 4:**
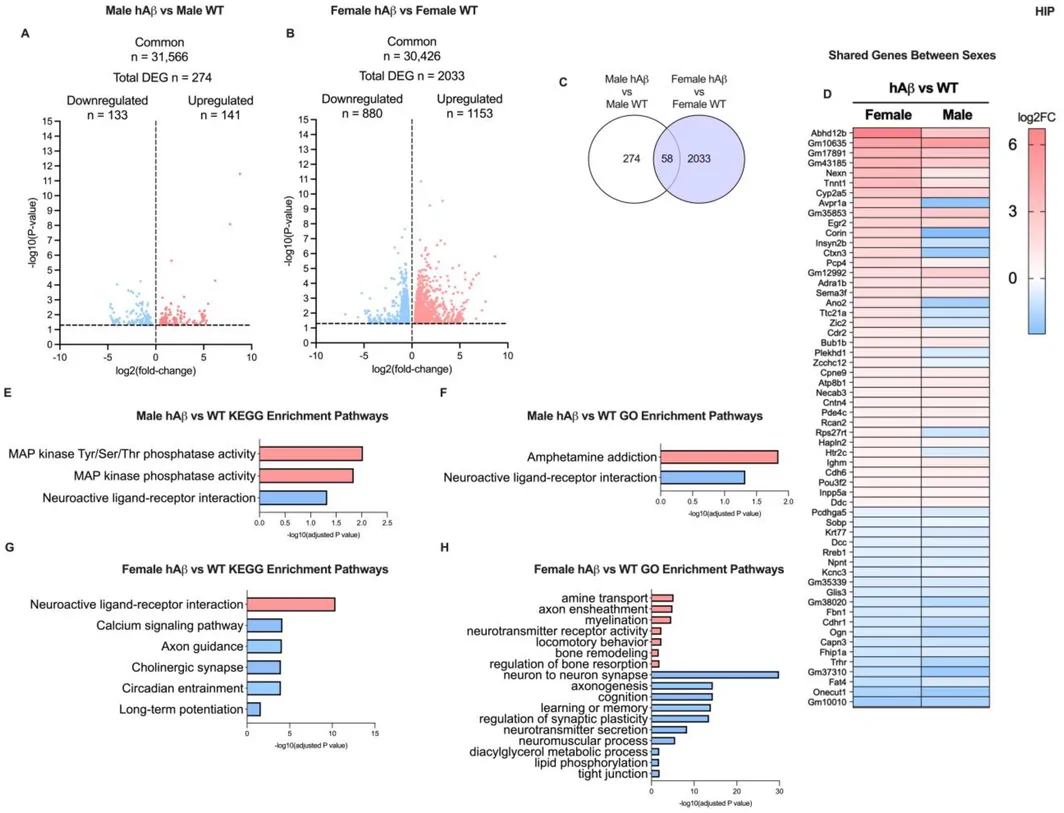
Differential gene expression and pathway enrichment in the hippocampus of male and female hAß‐KI compared to control mice. (A, B) volcano plots of differentially expressed genes (DEGs), upregulated is indicated in red, and downregulated in blue, calculated using log_2_ fold change (log2FC) and adjusted *p*‐value. (A) male hAß‐KI vs. male wild type (WT), and (B) female hAß‐KI versus female WT mice. (C) Venn diagram illustrating shared and sex specific DEGs, (D) Heatmap of shared DEGs between males and females shown as log2FC, a color scale included to indicate the direction and magnitude of each gene, (E) Male hAß‐KI compared to male control mice KEGG enrichment pathway, (F) Male hAß‐KI compared to male control mice GO enrichment pathway, (G) Female hAß‐KI compared to female control mice KEGG enrichment pathway, (H) Female hAß‐KI compared to female control mice GO enrichment pathway. All pathway analysis was calculated and displayed as −log10 (adjusted *p*‐value). There was an *n* = 3/sex/genotype, total *n* = 12.

To determine whether the hAß‐KI genotype alters transcription in a sex‐dependent manner, differentially expressed genes (DEGs) were analyzed separately in males and females (Figure 4A–C). In the hippocampus, the males had a total of 274 DEGs (141 upregulated, 133 downregulated), while the females had a total of 2033 DEGs (1153 upregulated, 880 downregulated). The hAß‐KI model resulted in a larger transcriptional response in the females than in the males, with ~9× more DEGs (Figure 4A–C). There were 58 genes of overlap indicating a sex‐specific transcriptional effect in the hAß‐KI (Figure 4C,D). Out of the 58 common genes, 11 of those between the male and female mice were not differentially expressed in the same direction (Figure 4D). The KEGG/GO enrichment pathways differed between the males and females as well. The males displayed an upregulation of the MAP kinase pathway activity with KEGG pathway analysis, and a downregulation of neuroactive ligand receptor interaction with the GO pathway analysis (Figure 4E,F). In the female KEGG enrichment pathway, a downregulation in calcium signaling and long‐term potentiation was observed (Figure 4G). In addition, as seen from the GO analysis, the females displayed an upregulation in locomotory behavior, bone remodeling, and regulation of bone resorption, and a downregulation in pathways related to cognition, memory, and the neuromuscular process (Figure 4G).

## 4 Discussion

This study provides a longitudinal characterization of male and female hAß‐KI mice across aging and identifies early metabolic risk factors that emerge prior to confirmed cognitive decline. An evident sex‐specific response was observed with the male hAß‐KI mice gaining more weight, losing lean mass, gaining fat mass at a faster rate, and exhibiting cognitive decline at 15 months, whereas the female hAß‐KI mice did not display these deficits. In addition, the male hAß‐KI mice displayed lower activity levels compared to their control counterparts and higher blood glucose levels in the ITT, indicative of insulin resistance. Most importantly, these temporal metabolic alterations occurred prior to the onset of cognitive impairment as confirmed by the 4‐h NOLT and NORT testing at 15 months. Our work not only aligns with previous work which confirmed that cognitive impairment was evident at 15 months of age [7], but together, our findings demonstrate the utility of the hAß‐KI mouse model for investigating metabolic risk factors associated with sAD and highlighting the importance of studying sex‐specific responses during disease progression.

Changes in body composition, such as the loss of lean mass and increased adiposity, have been identified as early metabolic changes that can contribute to AD risk [1, 16, 17]. For example, the prevalence of sarcopenia is significantly greater in individuals with AD compared to those who do not have AD [18, 19]. To our knowledge, this is the first comprehensive characterization of body composition changes in the hAß‐KI mouse model. While DXA analysis revealed increased adiposity, no significant differences were observed in the weights of three adipose depots collected at endpoint (Table S2A). This discrepancy is likely attributable to methodological differences, as the DXA allows for a sensitive measure of whole‐body fat, whereas endpoint tissue collection was limited to only three different adipose sites.

Taken together, these body composition changes indicate that metabolic dysfunction emerges before cognitive decline, thereby suggesting a temporal relationship between early metabolic dysregulation and cognitive impairment in the male hAß‐KI mice. However, these results do not determine a causal relationship. Previous research has determined that the male hAß‐KI mice exhibit higher insoluble fractions of Aß than females at 14 months of age [20]. The temporal emergence of metabolic dysfunction in the male hAß‐KI mice raises the possibility that these processes may be associated with the observed increase in amyloid pathology. However, additional studies are required to determine the causality of this relationship. Future studies should incorporate interventions targeted towards improving metabolic health (i.e., wheel running) to determine whether this impacts disease progression.

To gain further insight into the biological mechanisms, we examined transcriptional changes in the hippocampus and PFC using RNA sequencing. Previous RNA sequencing in the hippocampus and prefrontal cortex of the hAß‐KI mice at 22 months of age has been performed [7], however, these results were not differentiated by sex.

KEGG pathway analysis in the hippocampus demonstrated the upregulation of MAPK phosphatase activity in male hAß‐KI, suggesting a potential compensatory response to dampen MAPK regulation. This aligns with the established role of MAPK signaling in insulin resistance, obesity, and adipogenesis, all of which are seen in the hAß‐KI mice [21, 22]. Impaired insulin signaling is frequently associated with dysregulation of insulin receptor substrate (IRS)‐dependent pathways, resulting in altered activation of downstream signaling cascades including MAPK pathways [23]. In this context, the observed alterations in MAPK signaling may represent a molecular consequence of systemic metabolic dysfunction and provide a potential link between peripheral metabolic disturbances and AD‐related pathology. Furthermore, insulin can activate ERK, JNK, and P38 pathways which have all been linked to an increase in BACE‐1 expression and content, APP phosphorylation, neuronal apoptosis, brain atrophy, and cognitive impairment [23], supporting a mechanistic connection between the male‐specific metabolic vulnerability and AD‐related pathology. Further studies directly assessing brain insulin signaling and MAPK activation will be required to clarify the nature of this relationship.

In the PFC, KEGG pathway analysis revealed a sex‐specific regulation of mt‐Atp6 gene, a component of complex V in the mitochondrial electron transport chain [24] (Figure S1A, panel G). Mt‐ATP6 was downregulated in the male hAß‐KI alongside oxidative phosphorylation, and this result is consistent with other AD studies and could be indicative of mitochondrial dysfunction [24, 25]. In contrast, mt‐ATP6 was upregulated in the females, indicating a potential sex‐specific metabolic adaptation that needs to be further investigated.

In addition to the transcriptional changes observed in the brain, the hAß‐KI mice also exhibited evidence of potential systemic inflammation. Preliminary analysis of circulating immune factors from endpoint serum samples revealed that IL‐6 was elevated in the hAß‐KI mice compared to their control counterparts (Figure S2A). These findings suggest that inflammatory alterations may not be solely confined to the brain. Given the known link of IL‐6, insulin resistance, adiposity, and mitochondrial dysfunction, these findings suggest that peripheral inflammatory signaling may occur prior to the onset of cognitive impairment and could play a role in the initiation of AD [26]. However, the serum cytokine analysis including the PCA was performed as an exploratory analysis with a limited sample size, and therefore these findings should be interpreted cautiously. Future studies with larger cohorts will be required to validate these observations and to determine if peripheral inflammation precedes AD‐related pathology in the hAß‐KI mice.

While the male hAß‐KI mice exhibited evidence of metabolic dysfunction, the female hAß‐KI mice did not exhibit any differences in body mass, activity levels, or memory performance across the ages examined and they remained comparable to the female controls. Interestingly, the female hAß‐KI mice displayed greater insulin sensitivity compared to the controls, despite showing no changes in adiposity. This suggests a sex‐specific metabolic adaptation that needs to be investigated further.

The preservation of metabolic function in the female hAß‐KI mice prompted further examination of molecular pathways that may contribute to this adaptation. Notably, the female hAß‐KI mice exhibited a stronger transcriptional response in the hippocampus compared to males (2033 DEGs in females vs. 274 in males). The GO analysis in the female hAß‐KI compared to female control mice revealed a downregulation of pathways related to learning, memory, and cognition, as well as reduced expression of tight junction markers, suggesting a potential implication for the integrity of the blood‐brain barrier. Most notably, pathways associated with neuron‐to‐neuron synapses were downregulated, which is consistent with synaptic loss observed in aging and AD [27]. Despite the large number of transcriptional alterations, the female hAß‐KI mice remained protected from metabolic impairments, body composition changes, as well as cognitive impairment. This dissociation between the transcriptional response and phenotypic outcome suggests that the females may have a compensatory or protective mechanism that preserves their physiological function despite the observed transcriptional alterations.

One potential contributor to this preservation is estrogen, a well‐known neuroprotective hormone involved in the regulation of synaptic plasticity and neural survival [28, 29]. In addition, estrogen has been shown to reduce the production of Aß by inhibiting BACE‐1 expression [29, 30]. In humans, estradiol levels decline following menopause to concentrations lower than those in males [29]. Epidemiological studies reveal disparities, with a higher incidence of AD in postmenopausal females compared to males of similar age [30]. However, this sex‐specific disparity is not reported in our study because female mice do not naturally undergo menopause during aging [31]. The lack of a pronounced female phenotype should be interpreted cautiously, particularly given that women account for a substantial proportion of AD cases worldwide. Rather than suggesting reduced vulnerability, the current findings may reflect limitations of the experimental model, the age range examined, or species‐specific differences in disease progression. It is possible that female hAβ‐KI mice require additional aging, reproductive senescence, or metabolic stress to reveal pathological changes comparable to those observed in the males. Given the well‐established role of ovarian hormones in modulating metabolic health, future studies incorporating models of menopause, such as ovariectomy [32, 33], or accelerated ovarian failure [12], as well as metabolic challenges such as a high fat diet may help unmask AD‐related phenotypes in the female hAβ‐KI mice. Moreover, this would also allow for the exploration of the impact of estrogen on insulin sensitivity in the female mice. Prior to menopause, females are often protected from insulin resistance [34]. However, following menopause, there is an increase in dyslipidemia and body weight, alongside glucose intolerance, thereby indicating estrogen's potential role in glucose regulation [34].

Previous research around the hAß‐KI mouse model has largely focused on brain‐specific outcomes. This is the first study to our knowledge to characterize systemic risk factors relevant to sporadic AD. In addition, the longitudinal design for this study included sex‐specific analyses and assessment of body composition and metabolic outcomes at five different timepoints to identify when these alterations occurred. Limited studies have investigated sex‐specific responses with the hAß‐KI mice, with only one study demonstrating a sex difference in the insoluble fractions of Aß between male and female mice [20]. Our findings therefore highlight the need for sex‐specific investigation of AD pathology and known risk factors. However, some limitations should be acknowledged. First, the animals were maintained under single‐housing conditions, which may influence activity patterns, stress responses, and metabolic outcomes. To mitigate this, enrichment and nestling material were provided throughout the study. In addition, while the longitudinal design enabled the characterization of disease progression over time, mechanistic experiments were beyond the scope of the present study, and they will be required to establish a causal relationship between metabolic dysfunction and the subsequent cognitive decline reported in this study.

In conclusion, we demonstrated that the male hAß‐KI mice not only exhibited worse recognition and spatial memory but also displayed accelerated body composition changes and insulin resistance, thereby demonstrating that early metabolic alterations precede cognitive decline and may contribute to the development of sAD pathology in the male mice.

## Author Contributions

Conceptualization: E.M. R.E.K.M., and W.E.W. Methodology: E.M., R.E.K.M., W.E.W., V.A.F., and S.M.B. Investigation: E.M., E.L.C., J.G.S., A.A.T.M., and A.M. Visualization: E.M., R.E.K.M., V.A.F., and W.E.W. Supervision: R.E.K.M., W.E.W. and V.A.F. Writing (original draft): E.M., and R.E.K.M. Writing (review and editing): E.M. R.E.K.M., W.E.W., V.A.F, S.M.B., E.L.C., J.G.S., A.A.T.M., and A.M.

## Funding

This work was supported by the Brock University Explore Grant and the Brock University Chancellor Chair for Research Excellence (CCRE).

## Conflicts of Interest

The authors declare no conflicts of interest.

## Supporting information


**Table S1:** (A) Forward and reverse primer sequences used for hAß‐KI genotyping.

**Table S2:** (A) Relative endpoint adipose tissue weights for male and female hAß‐KI and control mice. Mean, standard deviation (SD) and *p*‐values for unpaired *t*‐test between the two groups of the same sex were reported.

**Figure S1:** (A) Differential gene expression and pathway enrichment in the prefrontal cortex of male and female hAß‐KI compared to control mice. (A, B) volcano plots of differentially expressed genes (DEGs), upregulated is indicated in red, and downregulated in blue, calculated using log_2_ fold change (log2FC) and adjusted *p*‐value. (A) volcano plot for male hAß‐KI vs. male wild type (WT), and (B) volcano plot for female hAß‐KI versus female WT mice. (C) Venn diagram illustrating shared and sex specific DEGs, (D) Male hAß‐KI compared to male control mice GO enrichment pathway, (E) Male hAß‐KI compared to male control mice KEGG enrichment pathway, (F) Female hAß‐KI compared to female control mice KEGG enrichment pathway, (G) Heatmap of shared DEGs between males and females shown as log2FC, a color scale included to indicated the direction and magnitude of each gene. All pathway analysis was calculated and displayed as ‐log10 (adjusted *p*‐value). There was an *n* = 3/sex/genotype, total *n* = 12.

**Figure S2:** (A) Serum PCA analysis for control and hAß‐KI mice. A PCA was performed on SPSS for the cytokines of IL1a, HGF, and IL‐6. A PCA was performed using a correlation matrix and z‐score variables. Prior to analysis, test for assumptions was performed such as KMO (0.597), Barlett's test of sphericity (7.84, *p* = 0.049) and determinant (0.335). The PCA extracted one component with eigenvalue of 2.11 (~70.2% of variance). (A) A *t*‐test was performed on factor 1 for control and hAß‐KI groups, (B) cytokine loading for factor 1 including IL1a, HGF, and IL‐6 are displayed with color scale ranging from −1 to 1 to indicate the magnitude and direction of each variable. There was an *n* = 2 for controls, *n* = 3 for hAß‐KI/sex, total *n* = 10.

## Data Availability

The data that support the findings of this study are available from the corresponding author upon reasonable request.
