# Supplementary material for: Metabolic Shifts Precede Cognitive Decline in the Male hAß‐KI Alzheimer's Mouse Model
Source: FASEB J. 2026 Aug 28;40(17):e72241. doi: 10.1096/fj.202602435RR (PMC13524197; doi:10.1096/fj.202602435RR)
Supplement: Supplementary file 1 [file FSB2-40-e72241-s002.pdf]

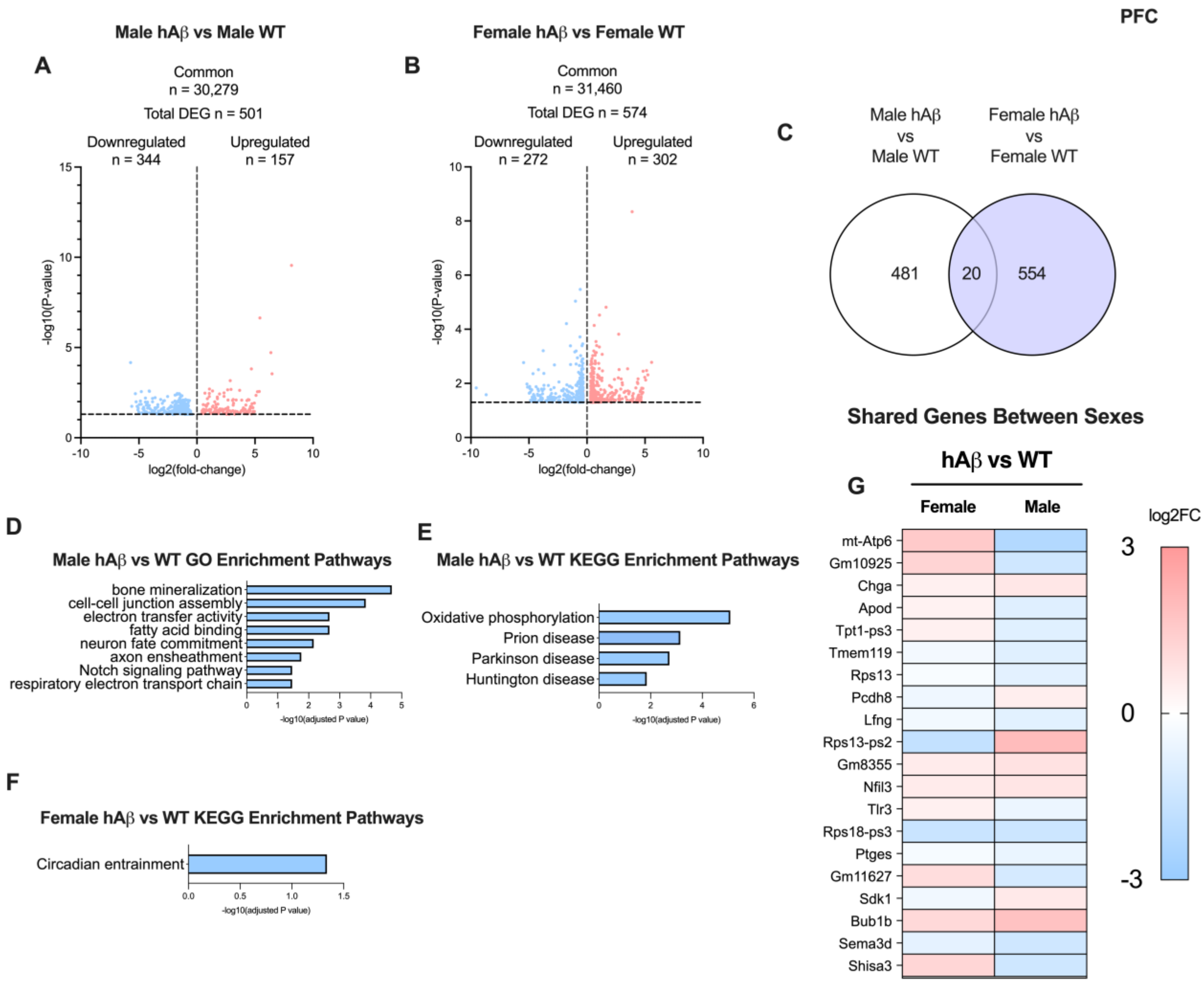

Figure 1A: Differential gene expression and pathway enrichment in the prefrontal cortex of male and female hAß-KI compared to control mice. (A-B) volcano plots of differentially expressed genes (DEGs), upregulated is indicated in red, and downregulated in blue, calculated using $\log_2$ fold change (log2FC) and adjusted p-value. (A) volcano plot for male hAß-KI vs male wild type (WT), and (B) volcano plot for female hAß-KI versus female WT mice. (C) Venn diagram illustrating shared and sex specific DEGs, (D) Male hAß-KI compared to male control mice GO enrichment pathway, (E) Male hAß-KI compared to male control mice KEGG enrichment pathway, (F) Female hAß-KI compared to female control mice KEGG enrichment pathway, (G)

Heatmap of shared DEGs between males and females shown as log2FC, a color scale included to indicated the direction and magnitude of each gene. All pathway analysis was calculated and displayed as -log10 (adjusted p-value). There was an n=3/sex/genotype, total n=12.
