# Supplementary material for: Metabolic Shifts Precede Cognitive Decline in the Male hAß‐KI Alzheimer's Mouse Model
Source: FASEB J. 2026 Aug 28;40(17):e72241. doi: 10.1096/fj.202602435RR (PMC13524197; doi:10.1096/fj.202602435RR)
Supplement: Supplementary file 2 [file FSB2-40-e72241-s004.pdf]

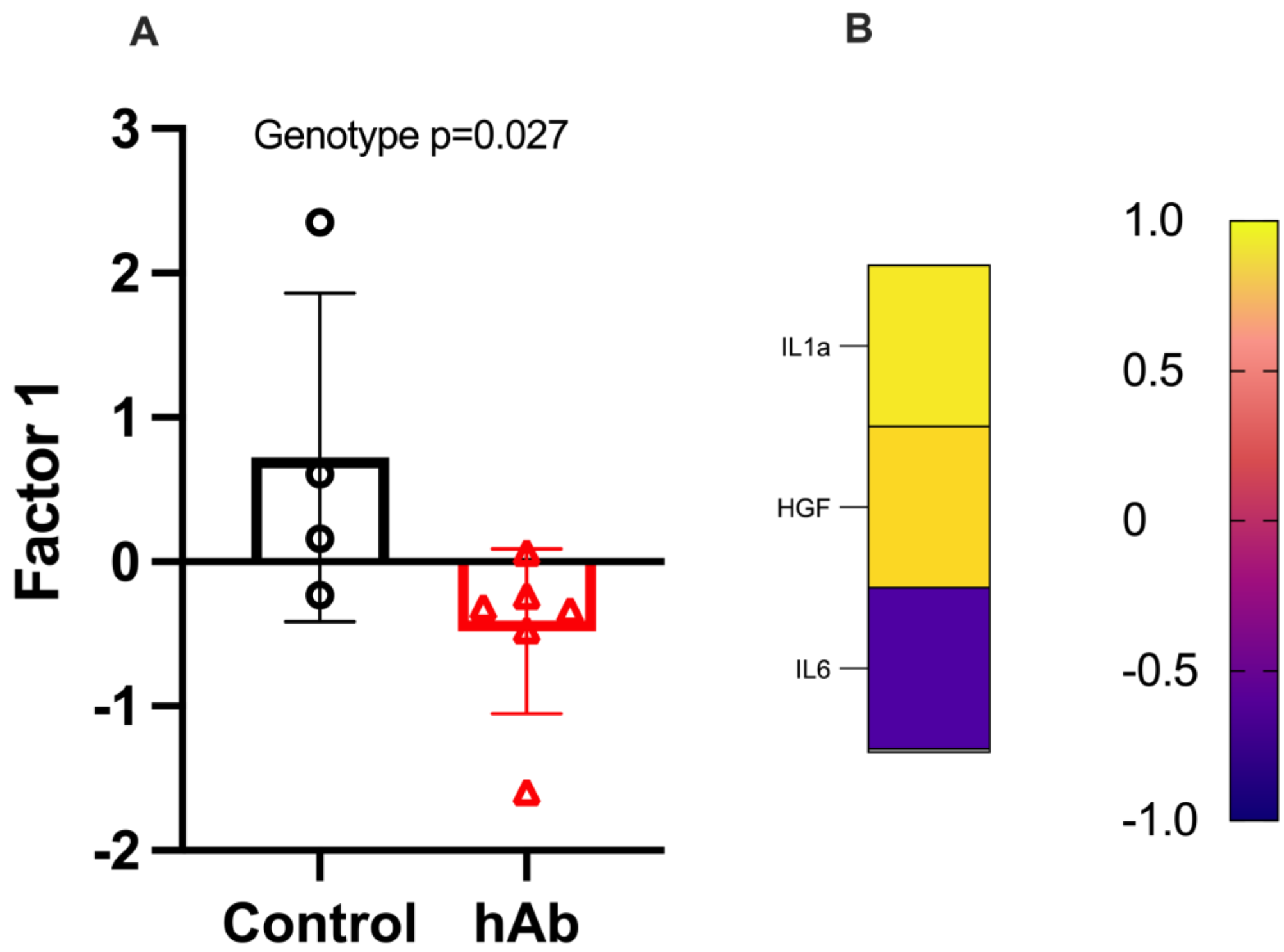


**Figure 2A:** Serum PCA analysis for control and hAß-KI mice. A PCA was performed on SPSS for the cytokines of IL1a, HGF, and IL-6. A PCA was performed using a correlation matrix and z-score variables. Prior to analysis, test for assumptions was performed such as KMO (0.597), Barlett's test of sphericity (7.84, p=0.049) and determinant (0.335). The PCA extracted one component with eigenvalue of 2.11 (~70.2% of variance). (A) A t-test was performed on factor 1 for control and hAß-KI groups, (B) cytokine loading for factor 1 including IL1a, HGF, and IL-6 are displayed with colour scale ranging from -1 to 1 to indicate the magnitude and direction of each variable. There was an n=2 for controls, n=3 for hAß-KI/sex, total n=10.
