# Supplementary material for: Metabolic Shifts Precede Cognitive Decline in the Male hAß‐KI Alzheimer's Mouse Model
Source: FASEB J. 2026 Aug 28;40(17):e72241. doi: 10.1096/fj.202602435RR (PMC13524197; doi:10.1096/fj.202602435RR)
Supplement: Supplementary file 3 [file FSB2-40-e72241-s001.docx]

|  | **Male Control** | **Male hAß-KI** | **p-value** | **Female Control** | **Female hAß-KI** | **p-value** |
| --- | --- | --- | --- | --- | --- | --- |
| Gonadal Adipose Tissue (gWAT) | 40.50  +/- 9.40 | 37.62  +/- 9.90 | 0.247 | 32.63  +/- 15.96 | 38.76  +/- 14.91 | 0.170 |
| Brown Adipose Tissue (BAT) | 4.24  +/- 1.14 | 4.39  +/- 1.11 | 0.372 | 3.77  +/- 0.77 | 3.71  +/- 1.07 | 0.440 |
| Inguinal Adipose Tissue (iWAT) | 29.10  +/- 12.89 | 31.11  +/- 12.91 | 0.356 | 28.09  +/- 8.72 | 31.31  +/- 11.36 | 0.221 |

**Table 2A.** Relative endpoint adipose tissue weights for male and female hAß-KI and control mice. Mean, standard deviation (SD) and p-values for unpaired t- test between the two groups of the same sex were reported.

*Tissue weight was adjusted for body weight by dividing tissue weight by body weight.
