# Supplementary material for: Metabolic Shifts Precede Cognitive Decline in the Male hAß‐KI Alzheimer's Mouse Model
Source: FASEB J. 2026 Aug 28;40(17):e72241. doi: 10.1096/fj.202602435RR (PMC13524197; doi:10.1096/fj.202602435RR)
Supplement: Supplementary file 4 [file FSB2-40-e72241-s003.docx]

**Table 1A**. Forward and reverse primer sequences used for hAß-KI genotyping.

| **Forward Primer** | CTG TGT GGG GGA AGC ATA AG |
| --- | --- |
| **Reverse Primer** | AAGA GCA TCC CTA CAA CCA AGT |
